# Rapid Molecular Diagnosis of Extra-Pulmonary Tuberculosis by Xpert/RIF Ultra

**DOI:** 10.3389/fmicb.2022.817661

**Published:** 2022-05-11

**Authors:** Laura Rindi

**Affiliations:** Dipartimento di Ricerca Traslazionale e delle Nuove Tecnologie in Medicina e Chirurgia, Università di Pisa, Pisa, Italy

**Keywords:** *Mycobacterium tuberculosis*, diagnosis, Xpert Ultra, extra-pulmonary, tuberculosis

## Abstract

Rapid detection of *Mycobacterium tuberculosis* complex and determination of drug resistance are essential for early diagnosis and treatment of tuberculosis (TB). Xpert MTB/RIF Ultra (Xpert Ultra), a molecular test that can simultaneously identify *M. tuberculosis* complex and resistance to rifampicin directly on clinical samples, is currently used. Xpert Ultra represents a helpful tool for rapid pulmonary TB diagnosis, especially in patients with paucibacillary infection. The aim of this review is to provide an overview of the diagnostic performance of Xpert Ultra in detection of extra-pulmonary tuberculosis.

## Introduction

Tuberculosis (TB) currently represents a major infectious disease worldwide. In 2019, the World Health Organization (WHO) estimated 1.2 million deaths and 7.1 million new cases (World Health Organization [WHO], 2020). Although TB mainly affects the lung parenchyma, *Mycobacterium tuberculosis* can spread to extra-pulmonary sites. On average, extra-pulmonary TB (EPTB) accounted for 16% of active cases of TB reported in 2019 (World Health Organization [WHO], 2020), with the proportion being greater among children and individuals positive for HIV. Pleurae and lymph nodes are the most common sites of involvement in EPTB, while meningeal TB, the most serious form of TB infection, occurs less frequently. EPTB diagnosis remains a great challenge because of the paucibacillary nature of the disease, subclinical or non-specific symptoms, and difficulties in obtaining qualified clinical specimens for *M. tuberculosis* detection.

In 2013 the World Health Organization (WHO) endorsed the use of Xpert MTB/RIF (Xpert) assay (Cepheid, Sunnyvale, CA, United States), a semi-automated cartridge-based molecular test, which allows for rapid TB diagnosis by simultaneous detection of *M. tuberculosis* complex and resistance to rifampicin (Boehme et al., 2010; Lawn et al., 2011; Theron et al., 2014; Detjen et al., 2015). However, the sensitivity of this assay is poor when the load of bacilli is very low and remains variable when tested on different types of extra-pulmonary specimens (Theron et al., 2014). Several systematic reviews and meta-analyses evaluated the performance of Xpert and reported pooled sensitivity ranging from 69 to 83% (Maynard-Smith et al., 2014; Penz et al., 2015; Jiang et al., 2020). Recently, the new version Xpert MTB Ultra (Xpert Ultra) has been developed to overcome the limitations of the Xpert assay (Chakravorty et al., 2017) and has been endorsed by the WHO in 2017. The Xpert Ultra system, based on the amplification of two multicopy sequences and characterized by improved assay chemistry, offers a greater sensitivity given the decreased limit of detection of *M. tuberculosis*. A prospective multicenter diagnostic accuracy study showed the excellent sensitivity of Xpert Ultra for detection of pulmonary TB, especially for paucibacillary specimens (i.e., from individuals with smear-negative TB or HIV infection) (Dorman et al., 2018). Similar results were found when the diagnostic performance of Xpert Ultra was evaluated for the detection of pediatric pulmonary TB (Sabi et al., 2018). Further studies confirmed that Xpert Ultra represents a useful tool for rapid diagnosis of pulmonary TB (Hodille et al., 2019; Kolia-Diafouka et al., 2019; Opota et al., 2019b; Ssengooba et al., 2020).

The Xpert Ultra assay has different sensitivities and specificities for detecting *M. tuberculosis* complex obtained from lymph nodes, pleural fluid, gastrointestinal tract, genitourinary system, cerebrospinal fluid, and other samples (Opota et al., 2019a; Zhang et al., 2020; Park and Kon, 2021). The aim of this review is to provide an overview of the current knowledge of the diagnostic accuracy of Xpert Ultra for the detection of EPTB, taking into consideration a broad range of different types of extra-pulmonary specimens. Recently, several studies have addressed the performance of Xpert Ultra for the diagnosis of EPTB, evaluating the diagnostic accuracy of Xpert Ultra compared with culture and/or a composite reference standard (CRS) based on clinical, laboratory, histopathologic, and radiological features. Table 1 summarizes the diagnostic performance of Xpert Ultra on EPTB specimens. The following paragraphs will consider the studies carried out on each of the main EPTB specimens.

**TABLE 1:** Diagnostic performance of Xpert MTB/RIF Ultra (Xpert Ultra) on extra-pulmonary TB (EPTB) specimens.

Specimen type	Sensitivity		Number of specimens		References
	vs Culture	vs CRS	Culture-Positive	Total	
Pleural fluid	84.2%	61.1%	57	108	Wang et al., 2019
	83.6%	44.2%	55	208	Wang et al., 2020
		43.7%		103	Wu et al., 2019
	47.6%		21	24	Perez-Risco et al., 2018
		37.5%		48	Meldau et al., 2019
	50%		6	118	López-Roa et al., 2021
	66.7%		9	77	Mekkaoui et al., 2021
	50%	48.1%	6	27	Gao et al., 2021
Pleural tissue	100%		2	2	Perez-Risco et al., 2018
	100%		2	41	López-Roa et al., 2021
	100%	81.48%	9	27	Gao et al., 2021
Lymph node tissue	94.1%		17	25	Perez-Risco et al., 2018
	75%		8	51	López-Roa et al., 2021
	95.8%		24	196	Mekkaoui et al., 2021
	90%	66.7%	10	24	Antel et al., 2020
		91.3%		46	Minnies et al., 2021
	50%		10	10	Bisognin et al., 2018
	100%	40%	1	5	Hoel et al., 2020
		33.3%		3	Piersimoni et al., 2019
Lymph node aspirate	77.8%	70%	9	30	Antel et al., 2020
	100%	26.7%	4	15	Hoel et al., 2020
CSF		44.2%		43	Wang et al., 2019
		33.3%		6	Piersimoni et al., 2019
	90%	95.4%	10	22	Bahr et al., 2018
	80%	63.6%	5	11	Chin et al., 2019a
		92.9%		42	Cresswell et al., 2020b
	90.9%	59.5%	22	42	Donovan et al., 2020
	96.4%	72%	56	204	Sharma et al., 2020
		46.7%		60	Shao et al., 2020
	71.4%	45%	14	76	| Huang et al., 2021
Joint fluid	87.5%		8	9	Perez-Risco et al., 2018
Osteoarticular pus	62.5%		8	8	Perez-Risco et al., 2018
	96.1%	90.9%	52	132	Sun et al., 2019
Osteoarticular biopsy	100%		2	21	Perez-Risco et al., 2018
	100%		3	27	Mekkaoui et al., 2021
Urine	100%		12	24	Perez-Risco et al., 2018
	100%		2	6	López-Roa et al., 2021
		18%^a^		84^a^	Minnies et al., 2021 ^a^
		17.2%^b^		203^b^	Andama et al., 2020 ^b^
		33.9%^c^		56^c^	Cresswell et al., 2020a ^c^
Gastric aspirate	75%		4	5	Perez-Risco et al., 2018
	50%		2	2	Bisognin et al., 2018
		60%		5	Piersimoni et al., 2019
	87.5%	60.5%	48	129	Sun et al., 2020
	85.4%	52.5%	48	141	Sun et al., 2021
Stool	80%		5	8	Perez-Risco et al., 2018
		60.3%		141	Sun et al., 2021
	83.3%		72	111	Kabir et al., 2021
		45.5%		126	Liu et al., 2021

*^a^TB lymphadenitis; ^b^pulmonary TB; ^c^TB meningitis.*

### Pleural Fluid/Tissue

Pleural TB is the most common type of EPTB in adults (World Health Organization [WHO], 2020). The diagnosis of pleural TB, depending on detection of *M. tuberculosis* in pleural fluids or biopsy tissues, is often challenging given the paucibacillary nature of the disease (Shaw et al., 2018). Recently, several studies have addressed the performance of Xpert Ultra for the diagnosis of pleural TB. In Wang et al. (2019) analyzed 108 pleural fluid specimens and showed an overall sensitivity of Xpert Ultra of 61.1%; the sensitivity was 84.2% vs. culture. In a larger multicenter cohort study, which included pleural fluids from 208 individuals diagnosed with pleural TB, the same authors reported a sensitivity of 44.2% against CRS and a sensitivity of 83.6% among pleural effusion culture-positive cases (Wang et al., 2020). This is in line with a further publication, where 43.7% of 103 pleural fluids were detected by Xpert Ultra (Wu et al., 2019). Perez-Risco et al. (2018) evaluated the diagnostic performance of Xpert Ultra on different types of extra-pulmonary specimens, including pleural fluids and biopsy tissues, demonstrating that 47.6% of culture-positive pleural fluids were detected by Xpert Ultra; this study also included 2 pleural biopsy samples, both detected by Xpert Ultra. A prospective observational study revealed a poor sensitivity (37.5%) of Xpert Ultra for the diagnosis of bacteriologically and/or histopathologically confirmed pleural TB; moreover, the authors demonstrated that pleural fluid concentration did not significantly improve the sensitivity of Xpert Ultra (Meldau et al., 2019). Another study, conducted in a high-resource setting with low TB prevalence, reported a sensitivity of Xpert Ultra compared with culture of 50% on pleural fluid samples, showing the lowest sensitivity among non-respiratory samples (López-Roa et al., 2021); as previously described (Perez-Risco et al., 2018), also in this case, the only 2 culture-positive biopsy tissues were detected by Xpert Ultra (López-Roa et al., 2021). A further study that assessed Xpert Ultra against culture in pleural fluid specimens found a sensitivity of 66.7% (Mekkaoui et al., 2021). Finally, in a most recent study, Xpert Ultra on 27 pleural fluid and 27 biopsy tissue samples yielded a sensitivity of 48.1 and 81.5%, respectively, showing that Xpert Ultra with pleural biopsy alone had a diagnostic capacity equivalent to that of pathological examination for pleural TB diagnosis. Moreover, the “trace” positive outcome of Xpert Ultra was highly supportive of TB diagnosis for both biopsy tissue and pleural fluid examinations (Gao et al., 2021).

For all the studies mentioned above, Xpert Ultra showed a specificity ranging from 95.1% for biopsy and 98.5% for fluid to 100%. When evaluated, rifampicin susceptibility results of Xpert Ultra were fully concordant with phenotypic results.

From the above data, it appears that Xpert Ultra, even if it showed a variable sensitivity in the different studies, has a great potential in the rapid diagnosis of pleural TB. In fact, when its outcomes were integrated into the CRS, an obvious increase in the percentage of patients with defined TB was observed. Moreover, pleural biopsy tissues provided higher yields than pleural fluids; however, the sensitivity of Xpert Ultra in pleural biopsy specimens should be accurately assessed in larger populations.

### Lymph Node Aspirate/Tissue

Tuberculosis lymphadenitis is a common extra-pulmonary manifestation of TB, both in low- and high- prevalence TB areas. Cervical lymph nodes are the most typical TB lymphadenopathy site (Qian et al., 2019). Perez-Risco et al. (2018) who evaluated the diagnostic performance of Xpert Ultra on 25 lymph node specimens with culture as the reference standard, showed a sensitivity of 94.1% and a specificity of 100%. Lower sensitivity of 75% vs. culture was reported in a multicenter study in a high-resource setting with low TB prevalence that included 51 lymph node tissues; the specificity was 95.3% (López-Roa et al., 2021). A recent large study, which assessed the performance of Xpert Ultra on 196 lymph node samples, reported a sensitivity of 95.6% and a specificity of 86.1% using a culture reference standard (Mekkaoui et al., 2021). Moreover, the diagnostic accuracy of Xpert Ultra for TB adenitis was prospectively evaluated on 99 adult patients from whom fine-needle aspirates (FNAs) and biopsy tissues were obtained using the CRS. Xpert Ultra sensitivity on FNAs was 70% and on tissues was 67% when compared with culture. Xpert Ultra on FNAs had a sensitivity of 78% and on tissues 90%; the specificity ranged from 96 to 100% using the CRS, and from 78 to 87% using culture as the reference standard. These findings support the use of Xpert Ultra on FNAs as an initial test when TB adenitis is suspected, as Xpert Ultra has a high sensitivity, is minimally invasive, and gives rapid results (Antel et al., 2020). More recently, Xpert Ultra has proved to be highly sensitive for the diagnosis of TB lymphadenitis in an HIV-endemic setting; this study reported sensitivity and a specificity of 91 and 76%, respectively, for 46 FNA biopsies using a culture and cytology reference standard (Minnies et al., 2021). Several other studies, which, however, analyzed a limited number of lymph node samples, demonstrated a significant diagnostic performance of Xpert Ultra in the detection of *M. tuberculosis* on lymph node specimens, supporting its use for the diagnosis of TB lymphadenitis, at least as an early test (Bisognin et al., 2018; Piersimoni et al., 2019; Hoel et al., 2020; Menichini et al., 2020).

### Cerebrospinal Fluid

Tuberculosis meningitis is the most serious type of EPTB, determining high mortality and severe disabilities (Manyelo et al., 2021). Among individuals positive for HIV, TB meningitis causes mortality of over 50% (Wilkinson et al., 2017). Rapid diagnosis, hampered by the paucibacillary nature of the disease and small volume of CSF, and adequate treatment are essential for the control of meningeal TB. Xpert Ultra is recommended as the initial diagnostic test for suspected TB meningitis, as confirmed in a prospective cohort study aimed to evaluate the performance of Xpert Ultra in 129 adults positive for HIV in Uganda: Xpert Ultra sensitivity was 70% for probable or definite TB meningitis, and it increased to 95% against CRS (Bahr et al., 2018). A further evaluation of Xpert Ultra sensitivity in meningeal TB by Wang et al. tested 43 CSF specimens and showed a sensitivity of 44.% and 86.36, using the CRS and bacteriological positivity as reference standards, respectively (Wang et al., 2019). In another study performed on 11 patients with TB meningitis, the sensitivity of Xpert Ultra was 63.6% (Chin et al., 2019a). In a following prospective validation study, including 51 participants with probable or definite TB meningitis, Xpert Ultra had 75.5% sensitivity; against the composite microbiological reference standard, Xpert Ultra had a sensitivity of 92.9%. The authors concluded that, despite the high sensitivity, Xpert Ultra cannot be used as a rule-out test given the negative predictive value of 93% (Cresswell et al., 2020b). Similar conclusions were reported by Donovan et al. (2020), who, however, described a lower overall sensitivity of 59.5% in diagnosing meningeal TB; in this prospective, randomized, diagnostic accuracy study, the sensitivity of Xpert Ultra against a reference standard of definite, probable, and possible TB meningitis was 47.2%. In particular, in patients negative for HIV, the sensitivity was 38.9%, while in patients co-infected with HIV, the sensitivity was 64.3%. A diagnostic accuracy study from India that assessed the performance of Xpert Ultra on 204 CSF samples reported an overall sensitivity of 72.05% (96.42 and 62.83% for definite and probable TB meningitis, respectively, where definite TB indicates cases in which *M. tuberculosis* was isolated from CSF culture and probable TB indicates cases that were culture/smearnegative but had a diagnostic score of >10), and a specificity of 100% (Sharma et al., 2020). A further prospective multicenter study was conducted in China on 60 individuals with symptoms suggestive of TB meningitis; using microbiological evidence as a reference, the sensitivity of Xpert Ultra was 93.3%. Xpert correctly identified 28 cases of TB meningitis, indicating an overall sensitivity of 46.7% (Shao et al., 2020). More recently, Xpert Ultra on 160 CSF specimens yielded a sensitivity of 45% for definite, probable, and possible TB meningitis and 81% for definite TB meningitis, thus suggesting that Xpert Ultra, outperforming culture, may speed up the diagnosis and appropriate treatment of patients (Huang et al., 2021).

The studies described above show variability in the sensitivity values of Xpert Ultra, probably also due to the different protocols used, such as volume of CSF specimens, fresh or frozen samples, and centrifugation or not of the sample. As shown in Table 2, the sensitivity of Xpert Ultra increases with the centrifugation step; on the other hand, sample volume does not affect the performance of Xpert Ultra. The identification of a standardized procedure should be a priority to improve the management of TB meningitis. A recent study described optimal procedures for the detection of *M. tuberculosis* in CSF using Xpert Ultra, recommending careful attention to the collection, handling, and processing of CSF to maximize the performance of Xpert Ultra (Chin et al., 2019b). However, the contribution of Xpert Ultra is extremely valuable for rapid diagnosis of TB meningitis, and its application as an initial test on CSF could represent an excellent diagnostic tool.

**TABLE 2:** Correlation between sensitivity of Xpert Ultra and cerebrospinal fluid (CSF) testing protocol.

Sensitivity		CSF protocol			References
vs Culture	vs CRS	Volume (ml)	Fresh/Frozen	Centrifugation step	
	44.2%	1	Fresh	No	Wang et al., 2019
90%	95.4%	0.5	Frozen	Yes (10 ml centrifugated and resuspended in 2 ml)	Bahr et al., 2018
80%	63.6%	2	Fresh	No	Chin et al., 2019a
	92.9%	0.5	Fresh	Yes (unknown ml centrifugated and resuspended in 2 ml)	Cresswell et al., 2020b
90.9%	59.5%	0.2	Fresh	Yes (6 ml centrifugated and resuspended in 0.5 ml)	Donovan et al., 2020
96.4%	72%	1	Fresh	No	Sharma et al., 2020
	46.7%	1	Fresh	No	Shao et al., 2020
71.4%	45%	1	Fresh	No	Huang et al., 2021

### Bone and Joint Fluid/Tissue

Diagnosis of osteoarticular TB is quite challenging given the low bacterial load present in joint and bone specimens and difficulty in obtaining specimens, since tubercle bacilli are present deep inside tissues. In Perez-Risco et al. (2018) analyzed several kinds of osteoarticular specimens, including 9 joint fluids, 3 paravertebral abscess aspirates, 5 osteitis abscess aspirates, 2 intervertebral disc biopsies, 4 bone biopsies, 3 synovial tissues, and 18 joint biopsies; the sensitivity of Xpert Ultra vs. culture ranged from 60% for osteitis abscess aspirates to 100% for the disc, bone, and synovial biopsies. In a study aimed at analyzing the diagnostic value of Xpert Ultra for osteoarticular TB in a high-burden setting, which included 132 osteoarticular pus specimens, sensitivities of 90.91 and 96.15 against the CRS and culture, respectively, were reported; when Xpert Ultra outcomes were integrated, the percentage of confirmed osteoarticular TB cases increased from 84 to 94%. Xpert Ultra showed a specificity of 100% and full concordance with the phenotypic test for the detection of rifampicin resistance (Sun et al., 2019). A recent study, which assessed the performance of Xpert Ultra on 27 osteoarticular samples, reported a sensitivity of 100% and a specificity of 87.5% using a culture reference standard (Mekkaoui et al., 2021). Therefore, Xpert Ultra was proved capable of detecting significantly more osteoarticular TB cases than culture, making it a helpful tool for rapid diagnosis of osteoarticular TB.

### Urine

Urogenital TB, responsible for 15– 40% of EPTB cases, is one of the most common forms of EPTB in both less and more developed regions (Figueiredo et al., 2017). The disease is often associated with delayed diagnosis and treatment, leading to serious consequences, such as renal failure. To date, only a few studies, which included urine in the analysis of extra-pulmonary samples, have evaluated the performance of Xpert Ultra on urine specimens for the rapid diagnosis of urinary tract TB. Perez-Risco et al. (2018) who evaluated the diagnostic performance of Xpert Ultra on 24 urine specimens with culture as the reference standard, showed a sensitivity of 100% and a specificity of 100%. A further study, which analyzed a small number of urine samples, again reported a 100% sensitivity of Xpert Ultra against culture (López-Roa et al., 2021).

Recently, there has been a growing interest in using Xpert Ultra on urine specimens to diagnose non-genitourinary TB. A first study aimed to evaluate the performance of urine Xpert Ultra for pulmonary TB reported low sensitivity (17.2%) but high specificity (98.1%), in reference to sputum-based testing. However, the sensitivity of Xpert Ultra was higher than TB-lipoarabinomannan (TB-LAM) assay and reached 50% in patients positive for HIV, with CD4 <100 cells/ml, thus suggesting that Xpert Ultra on urine samples could be an alternative approach for individuals with advanced HIV infection and unable to produce sputum samples (Andama et al., 2020). Moreover, urine Xpert Ultra has been used to diagnose disseminated TB in a case report, showing to be a useful adjunctive diagnostic tool for HIV-associated TB (Atherton et al., 2018). A further study determined the prevalence of disseminated TB by testing urine specimens with Xpert Ultra in adults positive for HIV with suspected meningitis. Urine Xpert Ultra was positive in 12% of the tested cohort, and in 41% of patients with definite TB meningitis, demonstrating that urine is an additional viable clinical specimen for use with Xpert Ultra. Despite the lack of concordance between Xpert Ultra and TB-LAM assay, which needs further investigation, the use of Xpert Ultra with urine could represent a rapid and non-invasive test for suspected TB meningitis and a prognostic tool in individuals positive for HIV with TB meningitis (Cresswell et al., 2020a). A recent study by Minnies et al. assessed the performance of Xpert Ultra on urine samples for the diagnosis of TB lymphadenitis: Xpert Ultra had a low sensitivity (18%) and a high specificity (98%) when tested on urine specimens compared to FNA biopsies; none of the patients negative for HIV had any positive urine Xpert Ultra, while 12% of the patients positive for HIV were urine Xpert Ultra-positive, thus indicating that the use of Xpert Ultra on urine from patients positive for HIV with presumptive TB lymphadenitis could reduce invasive sampling (Minnies et al., 2021). Therefore, urine Xpert Ultra as a tool to diagnose pulmonary or disseminated TB seems to offer good diagnostic usefulness for patients infected with HIV.

### Gastric Aspirate

Gastric aspirates represent an alternative type of specimen for the diagnosis of pulmonary TB in patients, especially children, who have difficulty producing sputum. Perez-Risco et al. (2018) found Xpert Ultra to be 75% sensitive for 4 culture-positive gastric aspirate specimens in adults. A multicenter accuracy study performed to evaluate the value of testing gastric aspirate with Xpert Ultra for diagnosis of childhood TB reported a sensitivity of 87.5 and 60.5% in bacteriologically confirmed TB and in total TB cases, respectively; in particular, the sensitivity was 80% in children aged <4 years, which is significantly higher than that in older children (48.1%). The specificity of Xpert ultra was 99.4%. In conclusion, the study showed that Xpert Ultra on gastric aspirate samples had a diagnostic value for the early and accurate diagnosis of TB, especially in younger children (Sun et al., 2020). More recently, the same authors analyzed the performance of Xpert Ultra on gastric aspirate specimens for the diagnosis of pediatric pulmonary TB in a high-burden area in China and reported an overall sensitivity of 52.5% and a sensitivity of 85.4% for 48 children with confirmed TB, concluding that gastric aspirate Xpert Ultra is an appropriate test for bacteriological TB confirmation in children (Sun et al., 2021).

### Stool

Microbiological confirmation of pulmonary TB in children with sputum is often difficult given the low volume and poor quality of specimens; induced sputa and gastric aspirates, obtained by invasive procedures, have a low diagnostic yield and may be inaccessible in low-resource settings. Stool samples are easily obtainable and represent promising alternatives to respiratory specimens for childhood TB. Recently, some stool processing methods for Xpert Ultra have been developed (Lounnas et al., 2020; Jasumback et al., 2021). Perez-Risco et al. (2018) found Xpert Ultra to be 80% sensitive for 5 culture-positive stool specimens in adults. A cross-sectional study on children aged <15 years from Bangladesh that assessed the performance of Xpert Ultra on stool to diagnose pulmonary TB in children reported a sensitivity of 83.3% (60 were positive out of 72 bacteriologically confirmed cases); a high proportion of Xpert Ultra positive assays had trace results (Kabir et al., 2021). More recently, Sun et al. (2021) analyzed the performance of Xpert Ultra on stool specimens for the diagnosis of pediatric pulmonary TB in a high-TB burden and resource-limited area in China, and reported an overall sensitivity of 60.3%. Among 48 children with confirmed TB, Xpert Ultra testing was equally sensitive on stool and gastric aspirate specimens (85.4%); however, the agreement between Xpert on stool and on gastric aspirate was moderate in children with active TB (Sun et al., 2021). A prospective cohort study revealed a 45.5% sensitivity of Xpert Ultra using stool specimens for the diagnosis of pulmonary TB in children; moreover, the authors demonstrated that stool sample-based Xpert Ultra was a comparable alternative to *M. tuberculosis* culture on respiratory samples (Liu et al., 2021). In conclusion, testing stool specimens with Xpert Ultra may provide a useful diagnostic tool for detecting pediatric pulmonary TB.

## Conclusion

Xpert Ultra represents the most recent advancement in the rapid molecular diagnosis of TB. Many studies have shown a potential for the use of Xpert Ultra in extra-pulmonary specimens. Xpert Ultra sensitivity differs by specimen type, with higher sensitivity among specimens obtained from lymph nodes (ranging from 50 to 100%) and CSF (ranging from 71.4 to 96.4%), and lower sensitivity when using pleural fluids (ranging from 47.6 to 84.2%). Moreover, the performance of Xpert Ultra proved to be particularly high in special populations, such as pediatric TB and HIV co-infected TB. In addition, the use of Xpert Ultra on extra-pulmonary samples, i.e., gastric aspirate and stool, has been demonstrated to be helpful for detecting pulmonary TB.

Although negative Xpert Ultra results are not sufficient to rule out TB, positive Xpert Ultra results may be useful in rapidly identifying EPTB cases, thus suggesting that Xpert Ultra is a useful rule-in rapid diagnostic test that can improve the definitive diagnosis of EPTB.

## Author Contributions

LR reviewed the literature and wrote the manuscript.

## Conflict of Interest

The author declares that the research was conducted in the absence of any commercial or financial relationships that could be construed as a potential conflict of interest.

## Publisher’s Note

All claims expressed in this article are solely those of the authors and do not necessarily represent those of their affiliated organizations, or those of the publisher, the editors and the reviewers. Any product that may be evaluated in this article, or claim that may be made by its manufacturer, is not guaranteed or endorsed by the publisher.
